# Comparison of capillary, venous and buffy coat blood samples in detecting *Plasmodium* species among malaria suspected patients attending at Hamusite health center. A cross-sectional study

**DOI:** 10.1186/s12879-021-06290-6

**Published:** 2021-06-15

**Authors:** Getu Abeje, Woyneshet Gelaye, Getaneh Alemu

**Affiliations:** 1 Department of Biomedcal Science, Samara University, Samara, Ethiopia; 2 Department of Medical Laboratory Science, Bahir Dar University, Bahir Dar, Ethiopia

**Keywords:** Capillary blood, Venous blood, Buffy coat, Malaria, Detection rate

## Abstract

**Background:**

Both capillary and venous blood samples have been interchangeably used for the diagnosis of malaria in Ethiopia. However, *Plasmodium* parasites are thought to be more concentrated in capillary than in venous blood. Hence, selecting a sample source where parasites are more concentrated is indispensable approach in order to maximize the accuracy of blood film microscopy. Therefore, the present study aimed to compare the detection rate and the parasitemia level of *Plasmodium* species from conventional capillary and venous blood films, and buffy coat preparations.

**Methods:**

A facility based cross-sectional study was conducted from Feburary to March 2020 among 210 febrile patients attending Hamusite health center, northwest Ethiopia. Capillary and venous blood samples were collected and buffy coat was prepared from each sample. Thin and thick blood films were prepared, stained, and examined microscopically following standard protocol. Data were analysed using Statistical Package for Social Sciences Software version 20 and Med-Calc software version 19.3.

**Results:**

Capillary blood buffy coat (61/210, 29.0%) had significantly higher detection rate as compared to capillary (48/210, 22.9%) and venous (42/210, 20.0%) blood films (*p* < 0.001). However, no significant difference was observed between capillary and venous blood films (*p* = 0.070) in detecting *Plasmodium* species. The highest and the lowest mean asexual stage parasite counts were found in capillary blood buffy coat (4692.88) and venous blood (631.43) films, respectively showing significant variations (*p* < 0.001). Mean gametocyte count was also highest in capillary blood buffy coat (3958.44). As compared to capillary blood buffy coat, the sensitivity of venous blood buffy coat, capillary blood film and venous blood film were 73.8, 78.7, 68.9%, respectively.

**Conclusion:**

Capillary blood buffy coat samples showed the highest sensitivity in detecting and quantitating malaria parasites that its use should be promoted in clinical settings. However, conventional capillary and venous blood films could be used interchangeably.

## Background

Malaria is a vector-borne disease caused by protozoan parasites of the genus *Plasmodium*. Four *Plasmodium* species; namely *Plasmodium(P) falciparum, P. vivax, P. malaria* and *P. ovale* commonly infect humans. A fifth species, *P. knowlesi*, has recently been reported to infect humans in Southeast Asia [1, 2]. *Plasmodium falciparum* and *P. vivax* are the most prevalent species in the world, and the former is the most virulent species in terms of both morbidity and mortality [3, 4].

Malaria is a global public health problem as it remains one of the most important causes of morbidity and mortality throughout the world [5]. However, most (90%) of the malaria cases occur in the African Region [6]. In 2018, about 228 million cases were reported globally with 405 thousand deaths with 67% of the deaths occured among under 5 years-old children, the most vulnerable groups. About 93% of the deaths were in Africa [5]. In Ethiopia, approximately 75% of the land mass, where 68% of the population lives, is malarious [7]. Despite considerable national efforts being made to reduce morbidity and mortality due to malaria in Ethiopia, the disease is still a prominent public health problem throughout the country [8].

Humans are primarily infected through the bite of an infected female *Anopheles* mosquito that inoculates sporozoites during blood meal [9]. After inoculation, parasites circulate with blood and those reaching to the liver undergo one cycle development. Then after, parasites infect and multiply inside red blood cells (RBCs) to bring the characteristic signs and symptoms [10]. The clinical manifestations usually appear within 10 to 15 days after the mosquito bite and includes fever, headache and vomiting. If not treated, malaria can become life-threatening by disrupting blood supply to vital organs [11].

In order to reduce the burden and public health impact of malaria, early diagnosis and prompt treatment of patients are key strategies. The diagnosis is made using different techniqes ranging from the simple rapid diagnostic tests (RDTs) to complex molecular methods. In routine diagnosis, microscopic examination of blood films (BFs) and multi-species RDTs are performed. According to the Ethiopian national malaria diagnosis and treatment guidline, all febrile patients visiting health posts should be tested using RDTs and patients visiting health centers and higher health institutions should be diagnosed with BF microscopy before prescribing anti-malarial drugs [7].

The detection of *Plasmodium* parasites via light microscopy either from capillary or venous blood is still the primary method in most health care facilities equiped with clinical laboratory [12]. According to the World Health Organization guideline, blood smears can be prepared either from capillary or venous blood samples [13]. On the other side, malaria parasites are thought to be more concentrated in peripheral capillary blood than in venous blood, making capillary blood more sensitive. In line with this, a few previous studies have concluded that capillary blood is more sensitive for the detection of *Plasmodium* species than venous blood [14, 15]. On the contrary, some other studies showed that there is no difference in both detection and parasitemia level among capillary and venous blood samples [16, 17]. Hence, more data from different endemicity settings is required in order to solve the discrepancy and influence policy makers so that more sensitive samples will be recommended for examination.

In addition, Ethiopia is moving from malaria morbidity control to elimination [18]. At this stage, occurrence of light infection with low parasitemia level is expected in endemic areas of the country. This, in turn, calls for use of more sensitive diagnostic techniques capable of detecting such light infections. Molecular techniques like polymerase chain reaction (PCR) are primary choices for this. However, PCR needs well trained personnel, complex laboratory, long time for result generation and it is costy. Hence, it is not feasible to apply PCR as a routine diagnostic technique in resource limited countries like Ethiopia. Maximizing the detection rate of blood film microscopy is an important alternative to tackle the existing problem. As part of this, collecting blood at the site where parasites are more concentrated or concentrating blood after collection (like buffy coat) should be promoted. Despite this, there is lack of data about variations in sensitivity of blood samples collected from capillary and venous sources as well as buffy coat (BC) concentrate in Ethiopia. Hence, the aim of the present study was to compare the detection rate of *Plasmodium* species from capillary, venous and buffy coat BFs among malaria suspected patients in Dera district, one of the known malarious areas in Amhara Region of Ethiopia.

## Methods

### Study design and area

A facility based cross-sectional study was conducted at Hamusite Health center, northwest Ethiopia from Feburary to March 2020. The health center serves for the people from Dera district. The district is located at geographic coordinates of 11° 43′ 0″ North and 37° 38′ 0″ East. The area is with an average altitude of 2077 m above sea level, mean annual rain fall of 1300 mm and mean annual temperature of 26 °C (source: District health office).

### Sample size calculation and sampling technique

This study was part of a project investigating ‘Epidemiology and associated factors of malaria in Dera district’. Prevalence of *Plasmodium* infection was considered to calculate sample size using the single population proportion formula. Previous prevalence (p) of 8.7% in a similar geographical setting [19], 4% margin of error (d) at 95% confidence level (Zα/2 = 1.96) and 10% of the initial sample size (19 participants) were assumed during sample size calculation. Hence, the final sample size was 210. Febrile patients who visited the health center laboratory for BF examination and met the eligibility criteria were recruited by systematic random sampling technique with sampling interval of 2. Febrile patients clinically suspected of malaria who were ≥ 5 years-old and volunteer to participate were included in the study. Individuals who took anti-malaria and antibiotics therapy with in four weeks before enrollment were excluded.

### Blood sample collection and processing

Two milliliters of venous blood was collected from the cubital vein and capillary blood was collected by finger puncture. Venous and capillary blood samples were collected from each participant simultaneously with time gap of less than 5 min. Thin and thick BFs were prepared using approximately 2 μl and 6 μl of blood, respectively. Furthermore, BC was prepared from both venous and capillary blood samples using heparnarized microhematocrit capillary tube. The capillary tube containing blood was centrifuged at 5000 rpm for 5 min. Then, the capillary tube was cut and the red cell layer just below the plasma (approximately 1–2 m meter) was aspirated using a micropipette and used for thin and thick BF preparatin. In this way, four BFs (from capillary blood, venous blood, capillary blood BC and venous blood BC) were simultaneously prepared from each participant and stained with 10% giemsa for 10 min and then examined microscopically following standard protocol. Asexual parasite stages and gamatocytes were counted separately from each positive smear against 200 and 1000 WBCs, repectively [20]. For quantitative report, slides from each sample source were read twice and the mean parasite count was reported as the final parasite load.

In order to ensure data quality, standard operating procedures were strictly followed and quality of Giemsa staining reagent was checked every week using known positive and negative blood samples obtained from Amhara Public Health Institute (regional reference laboratory). Moreover, slides were read by two laboratory personnel where each reader was blind to results of the other. Both malaria microscopists are certified for ‘malaria microscopy and quality assurance from the national medical parasitology reference laboratory’ and had more than two years of practical experience at health institutions in malaria endemic areas. Discrepant results were examined by malaria laboratory experts from Amhara Public Health Institute.

### Statistical analysis

Data were edited, entered, cleaned and then analyzed using Statistical Package for Social Science Software version 20. Descriptive statistics were manipulated to explain the study participants and to show the malaria prevalence and level of parasitemia from each BF preparation. Under nonparametric tests, ‘related samples McNemar test’ and ‘related samples Wilcoxon signed rank test’ were used to assess difference in parasite detection and parasitemia level, respectively between different BF preparations. ‘T-test for paired samples’ was used to calculate the mean difference in parasite count between capillary and venous blood sources. Sensitivity, specificity, Positive Predictive Value (PPV) and Negative Predictive Value (NPV) of capillary BF, venous BF and venous blood buffy coat results were calculated against that of capillary blood BC using Med-Calc software version 19.3. Kappa value (k) was also calculated to show the strength of test agreement between capillary BC results and other blood sources and preparations. Test agreements were declared as no agreement (k ≤ 0), none to slight (k = 0.01–0.2), fair (k = 0.21–0.4), moderate (k = 0.41–0.60), substantial (k = 0.61–0.80) and almost perfect (k = 0.81–1.00) [21]. Differences in parasite detection rate and mean parasite count across different BF preparations were considered significant if *p*-value ≤0.05 at 95% confidence level.

## Results

### Socio-demographic characteristics of study participants

Equal number of male and female participants, 105 each, were recruited in the study. Regarding to their age composition,153 (72.9%) and 57 (27.1%) participants were > 15 and 6–15 years-old, respectively. One hundred ninety three (91.1%) participants were rural residents while the remaining 17 (8.1%) were urban dwellers.

### Detection of *Plasmodium* species and Parasitemia level

Overall, 61 (29.0%) participants were confirmed to be infected at least by a single species of *Plasmodium* at least by one sample source or BF preparation. Regarding to *Plasmodium* detection by sample source, 61 (29.0%) and 45 (21.4%) participants were positive from capillary and venous blood sources, respectively. Capillary blood BC preparation was the most sensitive sample being positive in 61 participants while conventional venous BF was the least sensitive where parasites were detected in 42 (20.0%) participants (Table 1). The highest mean asexual stage parasite count was detected from capillary blood BC (4692.88 parasites/μl) while the lowest mean parasite count was from venous BF (2070.73 parasites/μl). Similarly, the highest gametocyte count was observed in capillary blood BC (3958.44 gametocytes/μl) as opposed to only 631.43 gametocytes/μl from venous BF (Table 2).

**Table 1:** Prevalence of *Plasmodium* species from capillary and venous blood sources among malaria suspected patients attending Hamusite Health center,northwest Ethiopia from February to March 2020

Variable	Total examined	Category	Number positive	Prevalence (in %)
Cummulative	210	Overall *Plasmodium*	61	29.0
		*P. falciparum* only	41	19.5
		*P. vivax* only	10	4.8
		Mixed infection	10	4.8
Blood Source	210	Capillary	61	29.0
		Venous	45	21.4
Capillary BF	210	Overall *Plasmodium*	48	22.9
		*P. falciparum* only	29	13.8
		*P. vivax* only	9	4.3
		Mixed infection	10	4.8
Venous BF	210	Overall *Plasmodium*	42	20.0
		*P. falciparum* only	23	11.0
		*P. vivax* only	9	4.3
		Mixed infection	10	4.8
Capillary blood BC	210	Overall *Plasmodium*	61	29.0
		*P. falciparum* only	41	19.5
		*P. vivax* only	10	4.8
		Mixed infection	10	4.8
Venous blood BC	210	Overall *Plasmodium*	45	21.4
		*P. falciparum* only	26	12.4
		*P. vivax* only	9	4.3
		Mixed infection	10	4.8
Parasite stage detected	61	Trophozoite only	44	72.1
		Schizont only	1	1.6
		Gametocyte only	2	3.3
		More than 1 stage	14	23.0
Gametocyte prevalence	210	Over all	16	7.6
		Capillary BF	15	7.1
		Venous BF	14	6.7
		Capillary blood BC	16	7.6
		Venous blood BC	15	7.1

Key to abrreviations: BF = Blood Film, BC = Buffy Coat

**Table 2:** Mean parasite count from capillary and venous blood samples among malaria suspected patients attending Hamusite Health center, northwest Ethiopia from February to March 2020

Parasite stages	Category	Number of cases	Minimum count	Maximum count	Mean count	SD
Asexual Stages	Capillary BF	47	80	26,000	2931.06	2320.00
	Venous BF	41	40	20,000	2070.73	1776.05
	Capillary Blood BC	59	40	30,000	4692.88	3217.24
	Venous Blood BC	43	120	28,000	3728.37	2483.90
Gametocyte	Capillary BF	15	200	4000	1177.33	512.62
	Venous BF	14	240	1680	631.43	228.87
	Capillary Blood BC	16	600	20,000	3958.44	2314.25
	Venous Blood BC	15	400	12,000	2096.67	1495.39

### Comparison of parasite detection across capillary and venous samples

Detection of *Plasmodium* species in capillary BC was significantly higher as compared to that of capillary and venous BF (*p* < 0.001). However, the difference in detection rate of parasites in capillary and venous BF was not statistically significant (*p* = 0.070) (Table 3). The mean asexual parasite count significantly varies across different sample sources and smear preparations. Similarly, mean gametocyte count between capillary blood BC and capillary BF (*p* = 0.032), capillary blood BC and venous blood BC (*p* = 0.005), capillary BF and venous BF (*p* = 0.041) were also significantly different (Table 4). We planned to compare the performance of each preparation using the combined result as a reference. However, we found that the detection rate of capillary blood BC was the same as that of the combined result. Hence, we used findings of capillary blood BC as a reference to evaluate the performance of the other blood film preparations. Accordingly capillary BF, venous BF and venous blood BC were with sensitivity of 78.7, 68.9 and 73.8%, respectively in detecting *Plasmodium* infection as compared to capillary blood BC results. The NPV of mentioned preparations were 92.0, 88.7 and 90.3%, respectively while the specificity and PPV were 100% for all preparations. However capillary BF,venous blood BC and venous BF had substantial to almost perfect test agreement with that of capillary BC result (kappa values of 0.84, 0.76 and 0.80, respectively). Species level analysis also showed that capillary BF, venous BF and venous blood BC had low sensitivity (70.7, 56.1 and 63.4%, respectively) in detecting *P. falciparum* mono-infection but higher sensitivity (90% for each preparation) in detecting *P. vivax* mono-infection. There was 100% agreement across all samples in detecting mixed infection (Table 5).

**Table 3:** Difference in detecting Plasmodium species across different sample sources and preparations among malaria suspected patients attending Hamusite Health center, northwest Ethiopia from February to March 2020

Comparison		P-value
Difference in detecting *Plasmodium* species between	Capillary BF and venous BF	0.070
	Capillary BF and capillary BC	0.000
	Capillary BF and venous BC	0.453
	Capillary BC and venous BF	0.000

**Table 4:** Mean difference in parasite count from capillary and venous blood samples among malaria suspecte patients attending Hamusite Health center, northwest Ethiopia from February to March 2020

Parasite stages	Comparison	MD	SD	SE	95% CI of MD	P-value
Asexual stages	Capillary Blood BC vs Capillary BF	2819.57	2659.08	775.73	1258.11–4381.04	0.001
	Capillary Blood BC vs venous BF	4354.63	2863.39	894.37	2547.04–6162.23	0.000
	Capillary Blood BC vs venous Blood BC	2478.14	2227.82	679.48	1106.90–3849.38	0.001
	Capillary BF vs venous BF	1277.50	870.4	275.25	720.77–1834.24	0.000
	venous Blood BC vs venous BF	1832.68	1559.83	487.21	847.10–2817.37	0.001
Gametocytes	Capillary Blood BC vs Capillary BF	2978.33	2418.87	1249.10	299.29–5657.38	0.032
	Capillary Blood BC vs venous BF	2695.00	2788.2	1546.62	− 674.79-6064.79	0.107
	Capillary Blood BC vs venous BC	2059.00	1181.37	610.06	750.56–3367.44	0.005
	Capillary BF vs venous BF	615.71	509.27	272.22	27.63–1203.80	0.041
	venous Blood BC vs venous BF	1586.43	1582.07	845.65	− 240.49-3413.35	0.083

Key to abbrevations: *MD* mean difference; *SE* standard error of the mean; *SD* standard deviation, *CI* confidence interval

**Table 5:** Performance of capillary blood film, venous blood film and venous blood buffy coat samples in detecting *Plasmodium* species among malaria suspected patients attending Hamusite Health center, northwest Ethiopia from February to March 2020

Parameter	Sample	Sensitivity	Specificity	PPV	NPV	Kappa value
In detection of overall *Plasmodium* infection	Capillary BF	78.7	100	100	92.0	0.84
	Venous BF	68.9	100	100	88.7	0.76
	Venous blood BC	73.8	100	100	90.3	0.80
In detection of *P. falciparum* mono-infection	Capillary BF	70.7	100	100	93.4	0.795
	Venous BF	56.1	100	100	90.4	0.673
	Venous Blood BC	63.4	100	100	91.8	0.736
In detection of *P. vivax* mono-infection	Capillary BF	90	100	100	99.5	0.945
	Venous BF	90	100	100	99.5	0.945
	Venous Blood BC	90	100	100	99.5	0.945
In detection of gametocytes	Capillary BF	93.8	100	100	99.5	0.965
	Venous BF	87.5	100	100	99.0	0.928
	Venous Blood BC	93.8	100	100	99.5	0.965

## Discussion

Malaria is still a puplic health concern in Ethiopia even though substantial decrease in malaria related mortality and morbidity due to large scale intverentions [8, 18]. Early and accurate case detection is essential in the fight against the disease which, in turn, requires examination of the right clinical sample using a sensitive diagnostic method. In the present study, we have compared the sensitivity of venous and capillary BFs as well as BC preparations from both sample sources for the detection and quantitation of *Plasmodium* species.

Over all parasite detection was higher from capillary blood than venous blood (29.0% vs 21.4%) which goes in line with findings from Cameroon where capillary and venous BFs detected parasites in 29.3 and 17.3% of the participants, respectively (*p* = 0.0109) [14]. The possible reason for the difference might be due to the ability of *P. falciparum* (the most prevalen species in Ethiopia and in the present study) infected RBCs to sequester in the endothelial cells lining capillary blood vessles. Hence, during finger pricking, the microvasculature where sequesterd parasites are located are direcly accessed [22]. On the contrary, this phenomenon will limit presence or abandence of parasites in the venous blood. Moreover, presence of parasite stages in peripheral blood greatly varies by time, especially in venous blood [3] that repeatedly collected samples are required before ruling out the infection in each of the sample sources [14]. However, in the present study, blood was collected only once due to logistic problems.

More specifically, the detection rate was much higher in BC concentrates of capillary blood. This is justifyable because parasitized RBCs easily float and concentrate in or slightly below the BC upon centrifugation [22]. This characteristic resulted higher parasitaemia levels in specimens prepared from BC samples than respective conventional BFs as supported by results of the present study. The present finding is in line with previous study results which showed 100% sensitivity of BC preparations [23, 24]. The difference in *Plasmodium* detection between conventional capillary and venous BFs was not significant (*p* = 0.070) in the present study, pronouncing the importance of BC preparation. However, the mean capillary BF parasitemia level was significantly higher than that of venous BF (*p* < 0.001) which was in line with findings from Gabon [15]. This result contradicts with reports from Cameroon [17, 20], Burkina Faso [25] and Ugand [16] where no significant difference in mean parasitemia level between capillary and venous BF examinations was noted. This might be due to the difference in sample size, population recruited, method of BF preparations and examination, and time of blood collection. For example, the study from Cameroon [17] collected data from asymptomatic children.

The sensitivity of capillary (78.7%) and venous (68.9%) BFs observed in the present finding was comparable to results reported from Gabon which was 81.5 and 73.4%, respectively [15]. A sensitivity gap of 9.8% observed between capillary and venous BF in the present study was also comparable with the sensitivity gap of 12% reported from Cameroon [14]. Such differences might be due to the inherent ability of *P. falciparum* infected RBCs to contain highly variable parasite proteins on their surface, which have the ability to express in capillary blood vessels leading higher parasitemia in capillary blood compared to venous blood. Moreover, infected erythrocytes that do not adhere to vascular endothelium cells have probability to become eliminated in the spleen which leads to lower parasitemia, and hence lower detection, in venous blood [26].

The sensitivity gap of 14.6% between capillary and venous BF in detecting *P. falciparum* mono-infection in the present study was slightly lower than results from a study conducted in Gabon with gap of 17.9% [15]. On the other hand, no such difference in sensitivity of capillary and venous BF was observed in detecting *P.vivax* mono-infection and mixed infections. There was almost perfect test agreement between the blood sources in detecting *P.vivax* and mixed-infection. *Plasmodium vivax* doesn’t have the characteristic to sequester in capillary vessels making no significant difference in parasite abundence between capillary and venous samples.

Capillary BF,venous BC and venous BF had sensitivity of 93.8, 99.5 and 93.8%; and NPV of 99.5, 87.5 and 99.0%, respectively in detecting *Plasmodium* gametocytes as compared to capillary BC with almost perfect test agreement (k > 0.80). Conventional capillary BF had more gametocyte parasite count than that of venous BF in the present study (*p* = 0.041). This difference might be due to the reason that capillary blood is utilized by the *Anopheles* mosquito and gametocytes are parasite stages infecting the insect vectors during blood meals. So, in order to perpetuate the life cycle, gametocytes might have higher tendency to be abundant in capillary blood. This was similiar with the study result in Gabon and Uganda where capillary BF had higher gametocyte count than venous BF [15, 16].

In addition, the sensitivity of capillary blood BC (100%) in the present study was higher than a sensitivity of 27.8% reported from Bankgkok [27]. On the contrary, the sensitivity of venous blood BC (73.8%) observed in the present study was lower than 95.8% sensitivity of venous blood BC reported from Cameron [22] and 100, 96 and 92.7% reported from three separate studies in India [28–30]. The reason for this difference might be due to differences in BC preparation procedure. We prepared BC in heparinized capillary tube by centrifuging for 5 min while separation of BC was made using wintrobe tube by centrifuging for 12–15 min in previous studies. In addition,we have used BC thick and thin films examined under 100x magnification with the aid of bright field microscope. In studies from India, quantitative BC preparation tube was used, and after centerifuging with high speed (12,000 rpm), it was examined with the aid of fluorescent microscope which increases the detection of *Plasmodium* species [26–28]. Similary sample size, local malaria prevalence and parasitemia level might affect the sensitivity. Further more, similar higher sensitivity results were reported from Thandalam [31]. In general, the present study showed that false negative results were higher in venous blood sample (9%) than in capillary blood sample with a sensitivity and parasitemia level gap of 31.1% and 2622.11, respectively. This finding facilitates use of capillary blood sample than venous blood sample not to miss light infections. The present study had limitations that we didn’t include asymptomatic study participants where light infections are expected. Moreover, performance of capillary and venous blood samples in detecting malaria parasites was evaluated using BF microscopy. Use of more sensitive molecular methods (like polymerase chain reaction) would help to give definitive conclusions.

## Conclusions

Use of capillary blood samples produces more accurate results than using venous blood samples in detecting malaria parasites. Capillary blood BC samples showed greater sensitivity in detecting *P. falciparum* mono-infection and in determing parasitemia level as compared to all other sample sources. Therefore, use of capillary blood BC should be promoted in clinical settings. We also recommend further studies assessing the sensitivity of capillary and venous samples in detecting *Plasmodium* species among asymptomatic individuals and by using more sensitive molecular techmiques.

## Data Availability

The original data for this study is available from the corresponding author.

## Abbreviations

BC: Buffy Coat
BF: Blood Film
NPV: Negative Predictive Value
PCR: Polymerase Chain Reaction
PPV: Positive Predictive Value
RBC: Red Blood Cell
RDTs: Rapid Diagnostic Tests
